# Universal screening for hyperbilirubinemia in term healthy newborns at discharge: A systematic review and meta-analysis

**DOI:** 10.7189/jogh.12.12007

**Published:** 2022-12-29

**Authors:** Faiza Khurshid, Suman PN Rao, Caroline Sauve, Shuchita Gupta

**Affiliations:** 1 Department of Pediatrics, Division of Neonatal-Perinatal Medicine, Queens University, Kingston, Ontario, Canada; 2 Department of Maternal, Newborn, Child, Adolescent Health and Aging, World Health Organization WHO; 3 Department of Education and Academy, Centre Hospitalier de l’Université de Montréal, Quebec, Canada

## Abstract

**Background:**

All term healthy neonates are screened for jaundice before hospital discharge as a standard clinical practice, but methods vary from clinical screening (visual inspection and/or risk factor assessment) to transcutaneous bilirubin (TcB) or total serum bilirubin (TSB) testing, depending on the setting.

**Methods:**

This systematic review of randomized and non-randomized studies evaluated the effectiveness of universal TcB and universal TSB screening at discharge compared to clinical screening alone for term healthy neonates. The outcomes were neonatal mortality, readmission for jaundice, severe hyperbilirubinemia (>20 mg/dL), jaundice requiring exchange transfusion, and bilirubin-induced neurological dysfunction (BIND). We searched MEDLINE via Ovid, EBM reviews, Embase, CINAHL, clinical trials databases, and reference lists of retrieved articles. Two authors separately evaluated the risk of bias, extracted data, and synthesized effect estimates using relative risk (RR) for randomized and odds ratio (OR) for non-randomized studies.

**Results:**

For universal TcB at discharge, we included one randomized trial enrolling 1858 participants and four non-randomized studies enrolling 375 956 participants. No study reported neonatal mortality. The randomized trial suggested that universal TcB at discharge may decrease readmission for jaundice (risk ratio (RR) = 0.24, 95% confidence interval (CI) = 0.13 to 0.46; low certainty evidence) and severe hyperbilirubinemia (RR = 0.27, 95% CI = 0.08 to 0.97; low certainty evidence), but the effect on jaundice requiring exchange transfusion (RR = 0.20, 95% CI = 0.01 to 41.6) and BIND (RR = 0.33, 95% CI = 0.01 to 8.17) was uncertain. Meta-analysis of non-randomized studies suggested that TcB may decrease severe hyperbilirubinemia (odds ratio (OR) = 0.25, 95% = CI 0.12 to 0.52; low certainty evidence) and jaundice requiring exchange transfusion (OR = 0.28, 95% CI = 0.19 to 0.42; low certainty evidence), but the effect on readmission for jaundice was uncertain (OR = 1.01, 95% CI = 0.38 to 2.7; very low certainty evidence). For universal TSB, we included three studies from the United States enrolling 490 426 participants. The effect on severe hyperbilirubinemia (OR = 0.37, 95% CI = 0.15 to 0.88), jaundice requiring exchange transfusion (OR = 0.53, 95% CI = 0.13 to 2.25) and readmission for jaundice (OR = 1.01, 95% CI = 0.62 to 1.67) was uncertain.

**Conclusions:**

Universal TcB at discharge may improve clinical outcomes for term healthy neonates. Evidence for universal TSB is uncertain.

**Registration:**

PROSPERO 2020 CRD42020187279.

Neonatal jaundice characterized by yellowish discoloration of the skin caused by hyperbilirubinemia affects up to 60% of term neonates and 80% of neonates with a gestational age of 35 weeks or more in the first two weeks after birth [1]. Globally, neonatal jaundice accounted for 1309 deaths per 100 000 live births and 113 401 disability adjusted life years (DALYs) in 2016 [2]. Severe hyperbilirubinemia, ie, serum bilirubin levels >20 mg/dL (342 μmol/L) is a leading cause of hospitalization in the first week of life and accounts for up to 35% of hospital readmissions in the first month of life [2]. If treatment is delayed, mortality, acute bilirubin encephalopathy, and bilirubin induced neurological damage (BIND) with neurodevelopmental delay and disability can occur.

It is important to systematically evaluate all neonates for jaundice before they are discharged from hospital to identify neonates who might be at risk of developing severe hyperbilirubinemia, as detection may be delayed or missed once the neonate is discharged [3]. While this is considered standard clinical practice and term healthy neonates are routinely screened for jaundice before hospital discharge in most settings, different screening methods may be used depending on the context [4]. In most low-resource settings, clinical screening (ie, visual inspection and risk factor assessment) is commonly practiced as the first step, followed by transcutaneous bilirubin (TcB) and total serum bilirubin (TSB) measurements as the second step if the clinical assessment suggests significant jaundice or risk [4,5]. In higher-income settings, TcB or TSB may be done for all neonates [5]. Available evidence suggests that clinical screening by visual inspection may not be accurate [6,7], and even experienced neonatologists may misdiagnose babies with jaundice [8]. Assessment of clinical risk factors also varies by setting and may not be optimal, especially in settings with high caseloads and early hospital discharge. However, TcB and TSB screening provide objective measurements of serum bilirubin levels. TcB is a non-invasive test which correlates well with serum bilirubin [9,10], while serum bilirubin is invasive, requiring a heel prick and laboratory assessment, but is an accurate and gold standard measure of hyperbilirubinemia.

We conducted this systematic review to ascertain the effectiveness of universal TcB screening at discharge or universal TSB at discharge compared to clinical screening (visual inspection and/or risk factor assessment) for improving neonatal outcomes.

## METHODS

This systematic review was registered in the PROSPERO database (PROSPERO 2020 CRD42020187279). We defined two study questions using the Patient, Intervention, Comparison, Outcome (PICO) framework:

- Study question 1: neonates (gestational age ≥35 weeks at birth) without complications (P); universal screening for jaundice by TcB (I); clinical screening (visual inspection and/or risk factor assessment) (C); neonatal outcomes (O).

- Study question 2: neonates (gestational age ≥35 weeks at birth) without complications (P); universal screening TSB (I); clinical screening (visual inspection and/or risk factor assessment) (C); neonatal outcomes (O)

### Types of studies

We included randomized (cluster-randomized or quasi-randomized) and non-randomized studies that compared universal TcB or TSB to clinical screening (visual inspection or risk factor assessment) in human neonates. We also planned to compare universal TSB with universal TcB if any study was found. Crossover trials were excluded. For both comparisons, ie, universal TcB vs clinical screening and universal TSB vs clinical screening, the intervention and the control groups could receive further investigations based on the initial assessment.

### Types of participants

Our review focused on term (≥37 weeks of gestation) healthy neonates, ie, neonates without any complications or illness during birth hospitalization. However, we did not exclude studies that enrolled late preterm neonates (ie, gestational age ≥34 weeks) or neonates with clinical risk factors for jaundice if they were otherwise well. Studies that enrolled neonates with visible jaundice or only preterm neonates were excluded.

### Types of interventions

Universal TcB or TSB was defined as TcB or TSB done for all neonates before discharge from hospital, irrespective of the presence of a clinical risk factor for hyperbilirubinemia. The TcB could be done using any standard TcB device, and TSB could be done using any standard laboratory method.

### Type of comparisons

The clinical screening could be visual inspection alone or combined with assessment of risk factors. This could be followed by TcB or TSB if required. The registered protocol on PROSPERO specifies two different comparisons as visual inspection alone (comparison 1) and risk factor assessment (comparison 2). We combined these two comparisons into one as “clinical screening” as we did not identify any studies that considered risk factors alone for screening purposes to determine whether or not a TcB or TSB would be done.

### Types of outcome measures

The studies must have reported at least one outcome of interest: neonatal mortality, hospital readmission for jaundice, severe hyperbilirubinemia (serum bilirubin level ≥20 mg/dL or 342.1 μmol/L), kernicterus or bilirubin-induced neurological dysfunction (BIND), jaundice requiring exchange transfusion, or neurodevelopmental outcome. We also recorded any reported side-effects of screening.

### Search methods for identification of studies

An experienced information specialist (CS) designed a comprehensive search strategy in consultation with the review authors. This strategy covered MEDLINE via Ovid, Cochrane CENTRAL, Embase, and CINAHL, updated by December 31, 2021. Clinical trials databases, reference lists of retrieved articles, and grey literature, including related conference proceedings (eg, Pediatric Academic Societies abstracts), were also searched for any eligible studies. The details of the search strategy are provided in the **Online Supplementary Document**.

The databases were searched independently by two review authors (FK and SR). Any discrepancies between the two reviewers were resolved by a third reviewer (SG). Searches were limited to human studies. There were no language restrictions.

### Data collection and analysis

Two authors independently extracted the data (FK and SR). Data extraction was done using a data extraction form that was designed and pilot tested by the review authors. The authors extracted information regarding design, methods, participants, interventions, outcomes, and treatment effects from each included study. We discussed disagreements until we reached a consensus. If data from the published reports were insufficient, their authors were contacted for clarification.

### Assessment of risk of bias

Two review authors (SG and SR) independently assessed the methodological quality of the included studies using the Cochrane risk of bias tool for randomized studies (RoB 2.0) and the risk of bias in non-randomized studies of intervention (ROBINS-I) tool for non-randomized studies [11,12]. Any disagreements between the review authors were resolved by mutual discussion.

### Statistical analysis

The meta-analysis was performed using RevMan (ver 5.4). Relative risk (RR) estimates for randomized and odds ratio (OR) estimates for non-randomized studies and their 95% confidence intervals (CI) were calculated when not provided by the study. If available, adjusted effect estimates provided by the studies were used. When the included studies provided only adjusted estimates without event rates in individual groups, the generic inverse variance method was used to calculate the pooled effect size and the participant numbers were entered manually where available.

We examined the heterogeneity between study results by inspecting the forest plots and quantifying the impact of heterogeneity using the *I^2^* statistic. If the *I^2^* statistic was <60%, we used the fixed-effect model; if the *I^2^* was >60% or *P* was <0.1, we explored the possible causes of heterogeneity. If there was no obvious clinical heterogeneity, we used the random-effects model for meta-analysis. We used the GRADE pro software to calculate the certainty of evidence. [13] We analysed and graded the evidence from randomized and non-randomized studies separately.

## RESULTS

**Figure 1:**
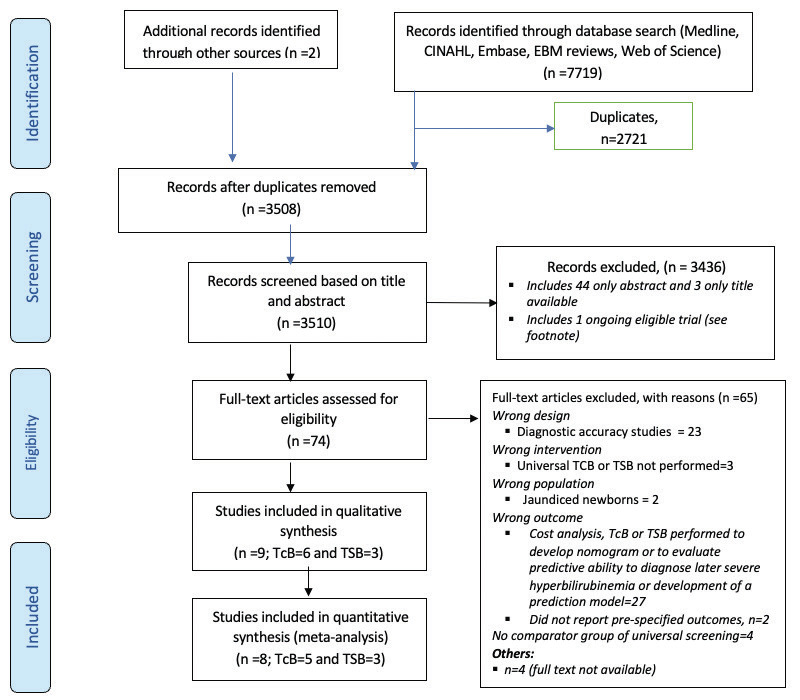
PRISMA flowchart for both comparisons.

Search results are shown in **Figure 1**. For comparison 1, ie, universal TcB screening, we identified a total of six studies that compared universal TcB at discharge with clinical screening. Of these, five studies were included in the final analysis, as one study performed daily TcB in hospital and post-discharge in the community and was summarized separately [14]. One large ongoing trial which seeks to compare universal TcB as the intervention was also identified [15]. For the second comparison, ie, universal TSB, three studies were identified that compared universal TSB with clinical screening.

The characteristics of the included studies are provided in **Table 1** (comparison 1) **Table 2** (comparison 2). The details of excluded studies are provided in table S1 in the **Online Supplementary Document**. One retrospective cohort study [19] contributed to both comparisons: it reported results from 11 hospitals, four of which adopted universal TcB screening policies, five adopted universal TSB screening policies, and two did not adopt either. The effect estimates for hospitals implementing universal TcB and TSB screening were reported separately. The effect estimates for the four hospitals using universal TcB screening and for the five hospitals using universal TSB screening were included in comparison 1 and 2, respectively. GRADE tables for both comparisons are available in the Table S2 in the **Online Supplementary Document**.

**Table 1:** Characteristics of included studies for comparison 1 – universal transcutaneous bilirubin at discharge vs clinical screening

**Okwundu, 2020 [** 16 **]**	
**Setting**	Hospital; Well-baby nurseries at Tygerberg Hospital, tertiary health care institution in Western Cape Province, Cape Town, South Africa.
**Study design**	RCT
**Participants**	Inclusion criteria: gestational age of at least 35 weeks and a birthweight of at least 1800 g, age ≤72 h old. Exclusion criteria: none specified.
**Intervention**	Pre-discharge TcB screening at the time of discharge followed by TSB if required. TcB level measured once pre-discharge using the JM-105 Jaundice Meter (Drager Medical, UK). An average of 3 readings was plotted on Bhutani’s hour-specific nomogram to determine the risk zone for hyperbilirubinemia. TSB was obtained for all infants in high-risk group to decide need for phototherapy while babies in high-intermediate-risk, low intermediate- risk and low-risk groups were asked to attend follow-up at a primary health care centre closest to their home at 24, 48, and 72 h, respectively, after discharge.
**Comparator**	Visual inspection at the time of discharge followed by TSB if required. The neonates were visually assessed for jaundice by admitting physician at the same time as TcB (at the time of enrolment); the presence of tissue yellowness was assessed by blanching the skin over the glabella and sternum. Venous blood samples for TSB measurement were only collected in infants who were clinically jaundiced or in those of rhesus D-negative mothers, according to the hospital protocol. The need for phototherapy was determined based on the TsB result. Neonates whose TSB value met the threshold for phototherapy according to the SA phototherapy guideline, were kept for such therapy before discharge home. Babies who did not meet the threshold for phototherapy and who were not visibly jaundiced at the time of discharge, were managed routinely. The mothers were asked to return with their neonates for follow-up assessments at the primary health care facility closest to their home within 2 d of hospital discharge.
**Outcomes**	Primary outcome: readmission for jaundice requiring phototherapy or exchange transfusion. Secondary outcomes: Phototherapy before hospital discharge, incidence of severe hyperbilirubinemia, critical hyperbilirubinemia or exchange transfusions and duration of hospital stay for those who were readmitted. Additionally, the rate of phototherapy before discharge was measured during hospitalization for the birth. Outcomes readmission for phototherapy, incidence of severe hyperbilirubinemia and duration of hospital stay were determined electronically through the database and by obtaining the infants files and electronic patient record transcripts from the hospital or primary health care centre where the baby was readmitted.
**Methods**	RCT. Allocation concealment: computer-generated allocation sequence sealed in sequentially numbered sealed opaque envelopes that were opened after informed consent was obtained. Blinding of treatment assignment: No blinding. Blinding of outcome measurement: blinded (outcome assessors and statisticians). Completeness of follow-up: Complete, no lost to follow up.
**Notes**	
**Alkalay, 2010 [** 17 **]**	
**Setting**	Well Baby Nursery at Cedars-Sinai Medical Center, UCLA, Los Angeles, California, USA.
**Study design**	Retrospective cohort
**Participants**	Neonates of gestational age of ≥34 weeks in the Well Baby Nursery.
**Intervention**	Universal neonatal hyperbilirubinemia screening in the Well Baby Nursery using transcutaneous bilirubin analyzer (BiliCheck, Philips Respironics, Marietta, GA). All neonates underwent screening for bilirubin prior to their discharge. July 1 2007 to June 30, 2008.
**Comparator**	Prior to 2003, no universal screening. Not clear if it was visual inspection alone or visual inspection along with consideration of clinical risk factors.
**Outcomes**	Readmission rates to the paediatric ward and the NICU of neonates with neonatal jaundice between the academic years 2002 and 2007.
**Methods**	Before and after study (retrospective)
**Notes**	N/A
**Flynn 2017 [** 18 **]**	
**Setting**	Hospital; Healthy newborn nursery of a community hospital in Fredericksburg, Virginia, south of Washington, DC.
**Study design**	Retrospective cohort
**Participants**	Inclusion criteria: all neonates greater than or equal to 35 weeks’ gestation admitted to the newborn nursery during the two separate six-week periods were included in the study. Exclusion criteria: Neonates with ABO incompatibility as confirmed with a positive Direct Antibody Test (DAT) test or Neonatal intensive care Unit (NICU) admission for greater than six hours of transitional care.
**Intervention**	All neonates before discharge had a risk assessment and screened with a TcB or TSB to determine the risk of developing severe hyperbilirubinemia. Neonates determined to have significant hyperbilirubinemia after being plotted on the AAP hour-specific nomogram were evaluated for the need for phototherapy or close primary care provider (PCP) follow-up. Neonates met criteria for phototherapy treatment based on the age in hours of life, risk factor assessment, and above or within 1 mg/dL below the AAP’s phototherapy nomogram level to treat. Neonates discharged before 72 h of life were required to have a discharge follow-up within 48 h. Neonates with risk factors for the development of hyperbilirubinemia were required to have a 24-h PCP follow-up or a bilirubin drawn within 24 h of discharge.
**Comparator**	Nurses randomly checked a newborn’s TcB when obtaining a metabolic screening test. A confirmatory TSB was required, per hospital protocol, on TcB in the high-intermediate or high-risk zones when appropriately plotted on the hour specific nomogram.
**Outcomes**	Total number of TSBs obtained on all neonates during their birth hospitalization, increase in “appropriate use of phototherapy”, Additional hospital days of stay.
**Methods**	A pre- and post-implementation study comparing two groups from two six-week periods. Retrospective chart reviews were conducted on the same six-week period in the years 2011 (pre-guideline) and 2012 (post-guideline).
**Notes**	
**Kuznewicz, 2009 [** 19 **]**	
**Setting**	Hospital: eleven hospitals of the Northern California Kaiser Permanente Medical Care Program, of which 4 adopted universal TcB screening policies, 5 adopted universal TSB screening policies, and 2 had not adopted either as of June 2007.
**Study design**	Retrospective cohort study
**Participants**	Neonates of gestational age of ≥35 weeks and birth weight were ≥2000 g in the Well Baby Nursery.
**Intervention**	Universal neonatal hyperbilirubinemia screening in the Well Baby Nursery using transcutaneous bilirubin analyzer (BiliCheck, Philips Respironics, Marietta, GA). All neonates underwent screening for bilirubin prior to their discharge; initiation of universal screening in October 2005. As reported in paper – bilirubin screening before discharge, with either TcB or TSB measurements. TcB measurements must be confirmed with TSB measurements if the TcB level is ≥15 mg/dL or if the TcB level plus 3 mg/dL is above the 2004 AAP phototherapy treatment line
**Comparator**	As reported by author by email correspondence – no universal screening, selective TSB (if indicated by visual inspection OR TcB).
**Outcomes**	Readmissions rates to the Pediatric Ward and the Neonatal Intensive Care Unit (NICU) of neonates with neonatal jaundice between the academic years 2002 and 2007.
**Methods**	Historical cohort study
**Notes**	The study does not report participant numbers separately for hospitals which adopted universal TcB screening but does report the adjusted odds ratio for the various outcomes of interest for the hospitals that adopted universal TcB screening.
**Wickeramasinghe, 2012 [** 20 **]**	
**Setting**	Hospital: level 1 (well-baby) nursery at the Rochester Methodist Hospital, Mayo Clinic, Rochester, Minnesota, USA
**Study design**	Retrospective cohort
**Participants**	Gestational age of ≥36 weeks discharged from well-baby (level 1) nursery
**Intervention**	Pre-discharge TcB screening for all infants using BiliChek (Respironics, Marietta, GA, USA) device. Before each measurement by a trained nurse. The BiliChek device was calibrated with a disposable tip (BiliCal). TcB values were adjusted by subtracting 1 mg/dL to obtain the optimal balance of sensitivity and specificity for screening. TcB values were then plotted on a web-based version (http://www.Bilitool.org) of the hour-specific serum nomogram described by Bhutani et al. to determine the risk for hyperbilirubinemia. Infants with adjusted TcB levels in the HIR (≥75th percentile) and HR (≥95th percentile) zones had a confirmatory TSB drawn, with subsequent TSB measurements obtained at the discretion of the treating health care provider. Infants with HIR and HR TSB values before hospital discharge had a repeat TSB ordered just before their first outpatient follow-up visit. Infants with adjusted TcB levels in the low-risk or low-intermediate risk zones were discharged without a TSB level unless there were other clinical risk factors for hyperbilirubinemia; these infants had outpatient TSB measurements only if deemed necessary by the treating outpatient provider.
**Comparator**	Clinical judgement of attending physicians
**Outcomes**	Not classified as primary and secondary. Overall – the number of bilirubin blood draws, the number of infants requiring phototherapy and the total newborn infant census for each month were obtained from medical record review.
**Methods**	Before and after study (retrospective)
**Notes**	All infants during periods 1 and 2 had follow-up outpatient visits scheduled between 2-5 d after discharge from the nursery. TcB measurement was not performed clinically in the outpatient setting.

RCT – randomized controlled trial, TSB – total serum bilirubin, TcB – transcutaneous bilirubin, g – grams, NICU – neonatal intensive care unit, HIR – high intermediate risk, HR – high risk

**Table 2:** Characteristics of included studies for comparison 2 – universal total serum bilirubin vs clinical screening

**Eggert, 2006 [** 21 **]**	
**Title of the study**	The Effect of Instituting a Prehospital-Discharge Newborn Bilirubin Screening Program in an 18-Hospital Health System
**Setting**	18-hospital health system, Intermountain Health Care, Salt Lake City, Utah
**Study design**	Historic cohort study. Involved all neonates delivered at ≥35 weeks’ gestation, within Intermountain Health Care’s 18-hospital system, during 2 periods of time: March 1, 2001, to December 31, 2002, and January 1, 2003, to December 31, 2004.
**Participants**	All neonates delivered at gestational age of ≥35 weeks
**Intervention**	A universal bilirubin screening program, TcB or TSB measurement on every neonate either at the recognition of clinical jaundice or before discharge, regardless of whether jaundice was observed: sixteen of the 18 hospitals used pre-discharge TSB. >99% of neonates had at least 1 pre-discharge bilirubin level measured if they were cared for in 1 of the 16 hospitals that screened for significant hyperbilirubinemia by quantifying serum bilirubin levels. For non-jaundiced neonates, the nursery staff was encouraged to obtain the screening TSB at the same time they obtained the state-mandated newborn screen for inborn errors of metabolism. TSB levels were measured in each hospital’s clinical laboratory as “bilirubin, neonatal” with Vitros 950 and 250 clinical chemistry analysers, (Ortho-Clinical Diagnostics, Rochester, New York State). Remaining two hospitals used pre-discharge TcB as primary method. These two hospitals used a screening jaundice meter for transcutaneous estimation of bilirubin levels (BiliChek; SpectRx Inc, Norcross, Georgia). Bilirubin values were plotted on an hour-specific bilirubin nomogram. If a bilirubin value plotted above the 40th percentile curve, the care provider was notified and intervention, evaluation, and follow-up was arranged as deemed necessary.
**Comparator**	The screening/management in pre-intervention group is not clear, but it was not universal screening as <20% neonates received TSB screening.
**Outcomes**	Readmission for jaundice; severe hyperbilirubinemia (≥20 mg/dL) and readmission to the hospital for treatment of hyperbilirubinemia. Bilirubin nomogram was modified in the initial months of the post-implementation period. “During the first months of the program, it seemed that an inordinately high number of neonates had bilirubin values in the intermediate- and high-risk zones of the Bhutani et al nomogram, 22 particularly when evaluated between 24 and 48 hours of age. Therefore, the 40th, 75th, and 95th percentiles of the hour-specific TSB values of the initial 4518 neonates screened between December 2002 and March 2003 were calculated and this population-specific data were used to modify the percentile tracks of the bilirubin nomogram. These modified percentile tracks were incorporated into the predictive nomogram of IHC’s bilirubin risk chart.”
**Bhutani, 2006 [** 22 **]**	
**Title**	A Systems Approach for Neonatal Hyperbilirubinemia in Term and Near-Term Neonates
**Settings**	Semiprivate urban birthing hospital.
**Study design**	Observational study. Included 41 961 live births at a large urban hospital from January 1, 1990, to December 31, 2000; 31 059 of these infants were discharged from the well-baby nursery as term and near-term healthy neonates.
**Participants**	Gestational age of ≥36 weeks discharged from the well-baby (level 1) nursery. 31 059 well babies discharged as healthy from a cohort of 41 961 live births (1990-2000). Term infants were defined as infants who were ≥38 weeks gestation. Near-term infants were those with <38 weeks gestation but had a BW of greater than or equal to 2000 g for 36 or more weeks gestation or BW≥2500 g for 35 or more weeks gestation. Exclusion criteria were: L BW preterm infants admitted to well-baby nursery and any infant admitted to and treated in the intensive care nursery for neonatal illness were excluded because of potential confounding effects on newborn jaundice and these infants do not meet the strict definition of a well-baby.
**Intervention** **and comparator**	Incremental implementation of a systems approach that incorporated a hospital policy to a) authorize nurses to obtain bilirubin (total serum/ transcutaneous) measurement for clinical jaundice, b) universal pre-discharge total serum bilirubin (at routine metabolic screening), and c) targeted follow-up, using the bilirubin nomogram (hour- specific, percentile-based total serum bilirubin/ transcutaneous bilirubin). Study phases: 1) Selective TSB measurement with MD orders (1990-1992). 2) Universal TSB (pre-nomogram)- unfettered nursing access to TSB orders; individualized lactation support (1993-1995). 3) Universal TSB (post-nomogram)- Lactation support program developed; nursing and parent education program for newborn jaundice (1996-1998). 4) Systems-based approach: implementation of all components (1999-2000). Phase 1 was considered as the “comparator” arm and Phases 2-4 above were considered as the “intervention” arm.
**Outcomes**	The study sought to determine the known adverse outcomes before, during, and after systems approach implementation. We used the outcome of hospital-based intensive phototherapy. This included either pre-discharge or readmission treatment. All study cohort babies had pre-discharge TSB levels obtained at the same time as the routine metabolic screen. In some, earlier TSB values had been obtained because of visual recognition or suspicion of jaundice. Hospital-based phototherapy was initiated at the discretion of the paediatrician. Unless modified for specific clinical reasons, guidelines for phototherapy published by the AAP (1994) were used
**Notes**	Implementation of the intervention took place as routine clinical practice. Data from two phases of implementation of universal screening was considered together in the current review
**Kuznewicz, 2009 [** 19 **]**	
**Setting**	Hospital: eleven hospitals of the Northern California Kaiser Permanente Medical Care Program (NCKPMCP), four of which adopted universal TcB screening policies, five adopted universal TSB screening policies, and two had not adopted either as of June 2007.
**Study design**	Retrospective cohort study
**Participants**	Neonates of gestational age of ≥34 weeks and BW was ≥2000 g in the Well Baby Nursery
**Intervention**	Universal neonatal hyperbilirubinemia screening in the Well Baby Nursery using TSB
**Comparator**	As reported by the author through email correspondence – no universal screening, selective TSB (if indicated by visual inspection OR TcB). As reported in paper – bilirubin screening before discharge, with either TcB or TSB measurements. TcB measurements must be confirmed with TSB measurements if the TcB level is ≥15 mg/dL or if the TcB level plus 3 mg/dL is above the 2004 AAP phototherapy treatment line.
**Outcomes**	Readmissions rates to the Pediatric Ward and the NICU of neonates with neonatal jaundice between the academic years 2002 and 2007.
**Methods**	Historical cohort study
**Notes**	The study does not report participant numbers separately for hospitals which adopted universal TSB screening but does report the adjusted odds ratio for the various outcomes of interest for the hospitals that adopted universal TSB screening.

RCT – randomized controlled tiral, TSB – total serum bilirubin, TcB – transcutaneous bilirubin, g – grams, BW – birth weight, MD – medical doctor, AAP – American Academy of Pediatrics, NICU – neonatal intensive care unit

### Comparison 1: Universal transcutaneous bilirubin at discharge vs clinical screening

#### Included studies

Five studies with 377 814 participants were included in the comparison of universal TcB at discharge compared to clinical screening alone. One study was a randomized trial from South Africa which enrolled 1858 participants [16] and four were retrospective cohort studies enrolling 375 956 participants from the United States. [17,18,20]. The studies were hospital-based and enrolled babies from well-baby nurseries.

#### Participants

All studies included a combined population of late preterm and term neonates. Three studies included neonates with GA≥35 weeks [16,18,19] and one study each included neonates ≥34 weeks. [17] and ≥36 weeks of gestation [20]. Two studies also had the additional criteria of birth weight (BW); Okwundu et al. included neonates with a BW≥1.8 kg and Kuznewicz et al. included those with a BW≥2 kg [16,19].

#### Intervention

All studies performed TcB screening before discharge on all babies [16,17,19,20]. In one study [18], TcB was done for all neonates at discharge (if it had not been done before) because of visible jaundice or the presence of clinical risk factors. The TcB was done using the BiliChek device (Respironics, Marietta, GA, USA) in two studies [17,20], the JM-105 Jaundice Meter (Drager Medical, UK) in one study [16], and two studies did not specify the device used [18,19].

The protocol for performing TSB following universal TcB screening varied across studies. One study obtained a TSB for all neonates in the “high-risk zone” based on an hour-specific TSB nomogram [16]. One study obtained a TSB on all infants at the time of the TcB [18], another if adjusted TcB (TcB levels minus 1 mg/dL) was in the high-intermediate risk (≥75th percentile) or high-risk (≥95th percentile) zones on the American Academy of Pediatrics (AAP)/Bhutani hour-specific nomogram [20]. One study obtained a TSB for TcB levels ≥12 mg/dL [17], and one if the TcB value was ≥15 mg/dL or within 3 mg/dL of the AAP phototherapy level [19].

#### Comparison

Clinical screening comprised visual inspection and/or risk factor assessment. The studies specified this variably as visual inspection (TSB was also obtained for babies with rhesus D-negative mothers) [16], “clinical judgment” [20], selective TSB (if indicated by visual inspection or TcB or positive Direct Antibody Test (DAT) in babies born to mothers with blood type O, Rh-negative or positive antibody screen) [19], personal communication with the author, and “random” TcB [18]. The study by Flynn et al. was included even though the comparison was a random TcB since it could still be considered a “non-universal” screening [18]. The comparison was unclear for one study, but it was not universal screening [17].

#### Risk of bias in included studies

A summary of the risk of bias assessment in the five included studies is depicted in Figure S1 in the **Online Supplementary Document** [16]. Some concerns of deviation from the intended interventions were noted in the RCTs, as blinding was not possible, and in the measurement of the outcome, as all neonates were not subjected to an objective evaluation for jaundice at readmission. Additionally, TSBs were done at the discretion of doctors at various primary health centres.

Three of the four non-randomized studies were at serious risk of bias [17,18,20] because they did not adjust for potential confounders. Two studies did not provide clear information on the denominators, and one had a potential deviation from intended interventions as universal TcB was done as part of routine clinical implementation and the intervention application could vary due to changes in clinical practices with time. One study was considered to be at high risk of selective reporting because it was a retrospective study and only one outcome was reported.

#### Outcomes

None of the studies reported the outcome of neonatal mortality. Readmission for jaundice was reported by all five studies. The outcomes of severe hyperbilirubinemia and jaundice requiring exchange transfusion were reported by two studies and was measured during readmission in one study [16] and in the first month after birth in the other. [19] One study reported BIND [16].

#### Effects of interventions

None of the included studies reported the outcome of neonatal mortality.

#### Readmission for jaundice

**Figure 2:**
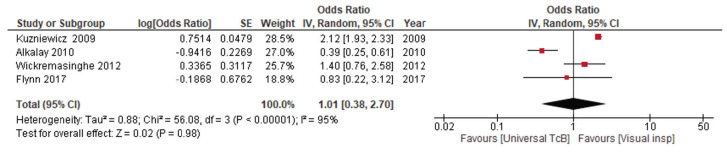
Forest plot for comparison 1 – universal transcutaneous bilirubin vs clinical screening-outcome readmission for jaundice.

One randomized trial reported that universal TcB at discharge reduced readmission for jaundice (1858 neonates; OR = 0.24, 95% CI = 0.13 to 0.46; low certainty evidence). A meta-analysis of non-randomized studies (**Figure 2**) reported that the effect was uncertain (four studies, 33 467 neonates; OR = 1.01, 95% CI = 0.38 to 2.70; very low certainty evidence).

#### Severe hyperbilirubinemia (serum bilirubin >20 mg/dl or 342.1 μmol/L)

One randomized trial found that universal TcB at discharge decreased the proportion of neonates with severe hyperbilirubinemia (one trial, 1858 neonates; RR = 0.27, 95% CI = 0.08 to 0.97; low certainty evidence) and non-randomized studies also found a reduction (one study, 358 086 neonates; RR = 0.25, 95% CI = 0.12 to 0.52; low certainty evidence).

#### Jaundice requiring exchange transfusion

One RCT reported that the effect was uncertain (one trial, 1858 neonates; RR = 0.20, 95% CI = 0.01 to 4.16; very low certainty evidence). However, the non-randomized study revealed that universal TcB at discharge reduced the proportion of neonates with jaundice requiring exchange transfusion (one study, 358 086 neonates; OR = 0.28, 95% CI = 0.19 to 0.42; low certainty evidence).

Only one randomized trial reported the outcome of kernicterus/BIND and the effect was uncertain (one trial, 1858 neonates; RR = 0.33, 95% CI = 0.01 to = 8.17; very low certainty evidence) [16].

Neurodevelopmental outcomes and side effects of screening were not reported in any of the included studies.

**Figure 3:**
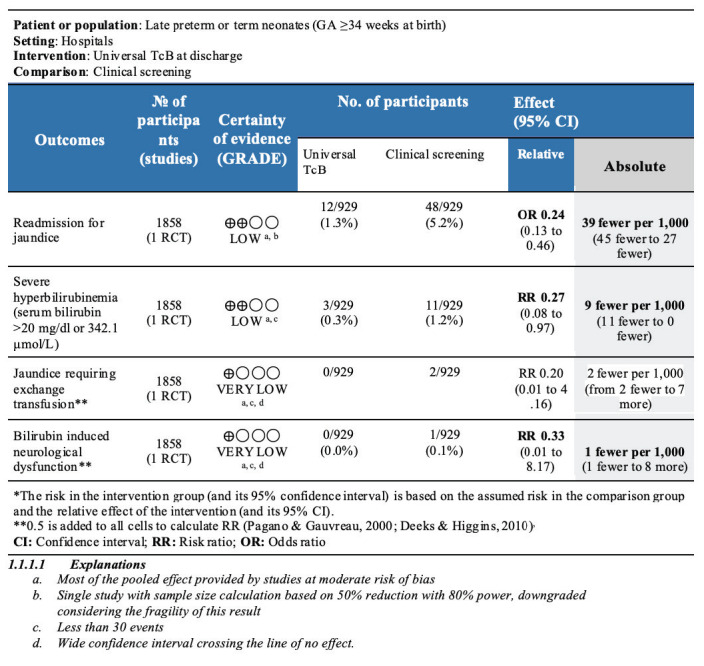
Summary of findings (SOF) for randomized study (comparison 1: universal transcutaneous bilirubin vs clinical screening).

**Figure 4:**
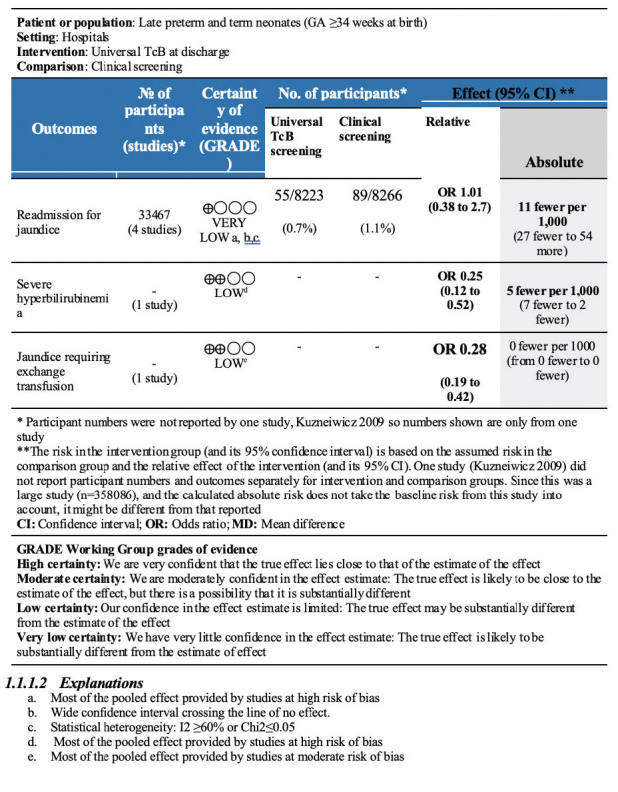
Summary of findings (SOF) for non-randomized studies (comparison 1: universal transcutaneous bilirubin vs clinical screening).

None of the studies provided a subgroup analysis for any outcome by gestational age to allow separate analysis for term neonates only. The summary of findings tables are shown in **Figure 3** (randomized studies) and **Figure 4** (non-randomized studies).

### Comparison 2: Universal transcutaneous bilirubin vs clinical screening

#### Included studies

For the comparison of universal TSB at discharge, two retrospective cohort studies [19,21] and one observational study [22] enrolling a total of 490 426 neonates were included. All were conducted in the United States.

#### Participants

All three studies included neonates with gestational age ≥35 weeks (490 426 neonates). Two studies also had birthweight as an additional criterion. Neonates were included if birthweight was ≥2000 g in one study (358 086 neonates) and ≥2000 g if born at ≥36 weeks, or ≥2500 g if born at ≥35 weeks (31 059 neonates). Two studies included all neonates discharged from the well-baby nursery [19,22] and one study included all live births [21].

#### Intervention

Two studies evaluated universal pre-discharge TSB screening done at the time of obtaining the state-mandated newborn screening for inborn errors of metabolism [21,22] while postnatal age at obtaining universal TSB screening was unclear in one study [19].

One historic cohort study [21] was conducted in a system with 18 hospitals, 16 of which did universal TSB (98 634 neonates), while two performed universal TcB screening (2647 neonates). The authors, however, indicated that the results obtained from the TcB measurements were not entered in the hospital system’s electronic database used for analysis. More than 99% of the neonates cared for in one of the 16 hospitals that practised universal pre-discharge TSB screening had at least one pre-discharge bilirubin level measured in the post-implementation period.

The observational study [22] reported data from four different periods: 1) selective TSB measurement with doctors’ orders (1990-1992), 2) universal TSB (pre-nomogram) – unrestricted nursing access to performing TSBs; individualized lactation support (1993-1995), 3) universal TSB (post-nomogram) – lactation support program developed; nursing and parent education program for newborn jaundice (1996-1998), and 4) systems-based approach: implementation of all components (1999-2000). For the current review, periods two, three, and four were considered as the “intervention” group. Universal TSB screening was done at the same time as routine metabolic screening. irrespective of the presence of clinical risk factors (<38 weeks, haemolysis, race/ethnicity, exclusive breastfeeding, family history, bruising/cephalohematoma).

The second retrospective cohort study (358 086 neonates) was conducted in eleven hospitals [19]; data for the five hospitals that practised universal TcB screening were included. The implementation of universal TSB screening was considered from the time when the facility achieved screening of ≥95% of all neonates.

TSB was measured using the 2,5-dichlorophenyldiazonium tetrafluoroborate (DPD) diazo method (Hitachi, 747, Boehringer, Mannheim Corporation Mannheim, Germany) in one study [22], the Vitros 950 and 250 clinical chemistry analysers (Ortho-Clinical Diagnostics, Rochester, New York State) in the second study [21], and is not described in one [19].

#### Comparison

In two studies, the comparison was clinical screening (visual inspection and/or clinical risk factor assessment). Selective TSB was done (selective meaning only for infants found to be at high risk on clinical screening) in one study [22], while the comparison was not clear in one study [21] but is likely to be clinical screening followed by selective TSB for those at risk, as <20% of neonates had a pre-discharge TSB done in the pre-implementation period compared to >99% in the post-implementation period.

#### Outcomes

Readmission for jaundice was reported in two studies [19,21]. Bhutani et al. also reported readmissions, but only during the various phases of “universal TS” intervention, therefore the data was not included [22]. Severe hyperbilirubinemia was defined at ≥20 mg/dL in one [21] and ≥25 mg/dL in the second study [19]. For jaundice requiring exchange transfusion, one study reported the odds of developing a TSB level over the 2004 AAP exchange threshold based on all TSB values from an infant’s first month of life [19]. The second study reported the incidence of exchange transfusion for the failure of intensive phototherapy during the first 7 days after birth [22].

#### Risk of bias in included studies

A summary of the risk of bias assessment in the three included studies is depicted in Figure S2 in the **Online Supplementary Document**. Two of the three studies were at serious risk of bias as they did not adjust the results for potential confounders. One study had a moderate risk of bias as the possibility of selective outcome reporting cannot be ruled out.

#### Effects of interventions

**Figure 5:**
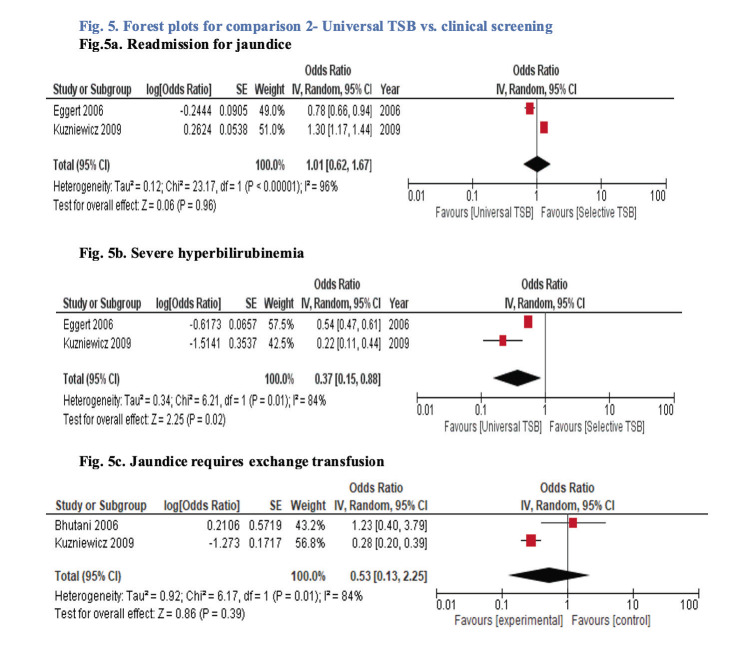
Forest plot for comparison 2 – universal total serum bilirubin vs clinical screening. **Panel A:** Readmission for jaundice. **Panel B:** Severe hyperbilirubinemia. **Panel C**: Jaundice requires exchange transfusion.

None of the studies reported neonatal mortality. The meta-analysis (**Figure 5**) suggested an uncertain effect for all three outcomes, ie, readmission for jaundice (OR = 1.01, 95% CI = 0.62 to 1.67; very low certainty evidence), jaundice requiring exchange transfusion (OR = 0.53, 95% CI = 0.13 to 2.25; very low certainty evidence), and proportion of neonates with severe hyperbilirubinemia (OR = 0.37, 95% CI = 0.15 to 0.88; very low certainty evidence). Neurodevelopment and side effects of screening were not reported by any of the included studies. No study provided subgroup analysis by gestational age for any outcome to allow separate analysis for term infants only.

One study [14] is summarized separately below. as it was not restricted to pre-discharge TcB. This study compared universal TcB with clinical screening using a before-and-after study design, enrolling 28 908 neonates. The study included all healthy neonates ≥35 weeks’ gestation in a well-baby nursery and who received universal TcB in the post-implementation period (daily TcB in hospital and post-discharge in the community) with visual inspection by a public health nurse in the pre-implementation period. The study reported that universal TcB decreased severe hyperbilirubinemia (OR = 0.45, 95% CI = 0.31 to 0.65) and readmission for jaundice (OR = 0.91, 95% CI = 0.81 to 1.04), while the mean length of pre-discharge hospital stay remained the same (40.8 ± 22.3 hours in universal TcB vs 40.3 ± 21.5 hours in the visual inspection group; mean difference = 0.5 higher, 95% CI = 0 to 1 higher).

**Figure 6:**
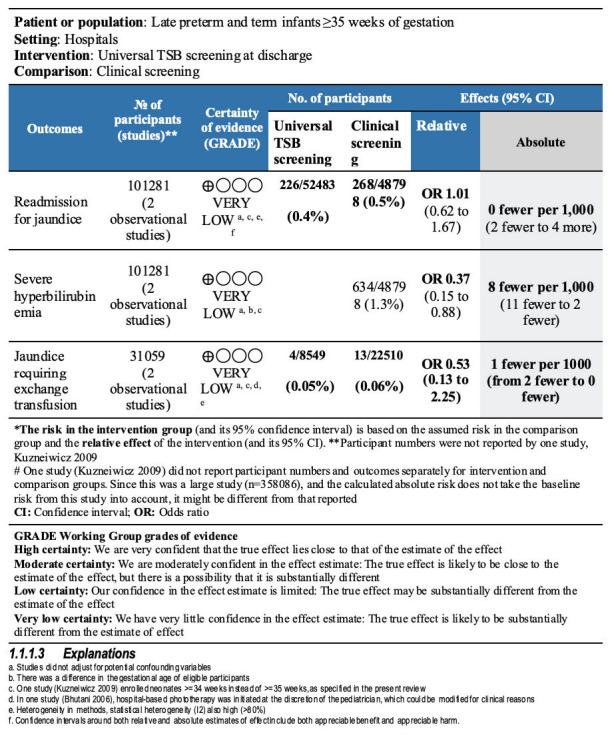
Summary of findings (SOF) for comparison 2 – universal TSB vs clinical screening.

The summary of findings table for comparison 2 is shown in **Figure 6**.

## DISCUSSION

Our review suggests that universal TcB at discharge for all term healthy neonates may reduce readmission for jaundice and severe hyperbilirubinemia but the effect on jaundice requiring exchange transfusion and BIND is uncertain. The effectiveness of universal TSB at discharge in improving neonatal outcomes is uncertain.

There is no previous systematic review evaluating the effectiveness of universal TcB at discharge or universal pre-discharge TSB for improving clinical outcomes of term healthy neonates. Few studies have reported clinical outcomes of babies assessed with universal TcB or clinical screening approaches, but available comparative studies report the benefit of TcB screening in identifying babies with higher levels of serum bilirubin [23] and reduced need for blood sampling and cost-effectiveness [24-26]. Many studies and a recent Cochrane review (Okwundu 2021, unpublished, personal communication with author) [27,28] show that TcB reliably estimates TSB levels in term neonates [29] with the sensitivity and specificity of TcB cut-off values to detect significant hyperbilirubinemia (TSB>95th percentile for age in hours) ranging from 74% to 100% and 18% to 89%, respectively [30]. There are concerns that TcB may overestimate TSB levels in babies with darker skin colour [31,32], but the evidence for this is conflicting [10].

Alternate risk assessment approaches such as visual assessment have been shown to be less accurate and reliable (r = 0.37 to 0.74) compared with the gold standard of laboratory serum bilirubin measurements [10]. Clinical risk factors alone or in combination with universal TcB screening have also been recommended as an alternative approach by many professional organizations, including the AAP [5,33]. The risk of severe hyperbilirubinemia may be considered low if no risk factors are present and may increase with the presence of one or more risk factors [33]. However, it is difficult to make an objective assessment, as multiple risk indices or clinical risk factor scoring systems have been shown to have variable predictive performance when compared to a pre-discharge TSB [34,35]. A risk prediction tool constructed using TcB and gestation for predicting subsequent significant hyperbilirubinemia showed that TcB alone compared well with the combination of TcB and risk factors, with the area under the curve (c-statistic) being 0.72 for pre-discharge TcB alone compared to 0.58 for clinical risk factors alone and 0.75 for the combination [3]. While universal TcB may be better than clinical screening alone for pre-discharge risk assessment, the role of routine clinical monitoring of neonates for the development of jaundice during health facility stay and measurement of serum bilirubin in those at risk (eg, in neonates with jaundice on the first day after birth) remains critical for in-patient management during a birth hospital stay.

A systematic assessment for jaundice at the time of discharge helps identify at-risk neonates, thus providing an opportunity for preventive/therapeutic interventions to be initiated in a timely manner with the potential to reduce the number of neonates needing readmission and/or developing neurotoxic levels of bilirubin. Hence, such an assessment is recommended as essential for discharge readiness [36]. The importance of universal screening at discharge may be understood by the fact that even in a high resource setting like Sweden, a 2016 study [37] reported the incidence of kernicterus as 1.3 per million live births; among the 13 children who developed kernicterus, brain injury was potentially avoidable for 11 children and an untimely discharge or a discharge with a lack of pre-discharge bilirubin screening was the possible cause in six babies (46%). While all professional organizations recommend a systematic assessment of neonates for jaundice before discharge, the methods vary. A recent meta-analysis of published guidelines reported that the United Kingdom, Italian, and Norwegian jurisdictions recommend visual inspection as a primary measure while others like Canada, the United States, Israel, and Turkish paediatric societies have endorsed universal screening [5]. The protocols for performing TSB based on TcB levels may vary from unit to unit but high-risk neonates are unlikely to be missed.

Our review highlights several important research gaps. There is no clear answer as to whether universal screening for hyperbilirubinemia using TcB or TSB reduces adverse outcomes such as acute and chronic encephalopathy or neurodevelopmental impairment, because of the low incidence of these morbidities and multiple factors concerning management and follow-up after the screening. There is a need for adequately powered trials comparing the effectiveness of various screening strategies on clinically relevant neonatal and infant outcomes.

Our review systemically evaluates the effectiveness of common approaches for screening well neonates for jaundice at discharge for clinically relevant outcomes. We took a comprehensive approach and evaluated evidence from both randomized and non-randomized/observational studies using standard, methods.

Our review has some limitations. First, while it is known that gestational age is an important risk factor, we could not perform a subgroup analysis for term healthy neonates [38] alone, as we were limited by the available data in the included studies. Second, the intervention of TcB or TSB was performed using any device or any standard method, even though the inter-device variability of TcB has been shown to be substantial [39] and there could be variability in TSB analysed using different methods (such as dry chemistry or diazo methods) [40]. The studies also used different nomograms and thresholds to perform TSB following the TcB assessment, which could impact the results [41]. The comparison of clinical screening was not well described in many studies and it was not clear whether it involved visual inspection alone, clinical risk factor assessment alone, or both. However, our review highlights that the clinical screening approach may not be consistent across settings and may vary over time. There was little data on important clinical outcomes such as neonatal mortality, severe morbidity including BIND/kernicterus, neurodevelopmental delay and adverse outcomes of screening. Lastly, our review was focused on approaches for hyperbilirubinemia screening at discharge, but this is only one variable in improving neonatal outcomes. The importance of a post-discharge follow-up program in decreasing the incidence of hyperbilirubinemia, especially for babies who are discharged >72 hours after birth, is well-recognized [42].

We did not provide an evaluation of the economic impact of universal screening. It is clear that any screening program using TcB and TSB will have an associated cost and the burden of disease will be highest in low- and middle-income countries (LMICs) where health budgets are limited. The review highlights the importance of timely diagnosis to health care professionals and facilities so that resources can be utilized efficiently. This point was recognized as one of the recommendations in the 2015 Don Ostrow Trieste Yellow Retreat (DOTYR-1) session devoted to LMICs [43].

Our findings are applicable to late preterm and term healthy neonates, ie, those who have had an uneventful birth hospitalization with no complications and are otherwise clinically well. Neonates who have visible jaundice were excluded, as they have a high probability of receiving TcB or TSB.

## CONCLUSIONS

Our review suggests that universal TcB screening at discharge should be part of the neonatal discharge readiness assessment for all facility births. Facilities implementing universal TcB screening should be able to obtain definitive testing (ie, serum bilirubin) and provide appropriate treatment and follow-up for all neonates, with ongoing monitoring for quality of implementation. The effectiveness of universal pre-discharge TSB screening in improving neonatal outcomes is uncertain.

## Additional material


Online Supplementary Document
