# Supplementary material for: Universal screening for hyperbilirubinemia in term healthy newborns at discharge: A systematic review and meta-analysis
Source: J Glob Health. 2022 Dec 29;12:12007. doi: 10.7189/jogh.12.12007 (PMC9798347; doi:10.7189/jogh.12.12007)
Supplement: Online Supplementary Document [file jogh-12-12007-s001.pdf]

# SUPPLEMENTARY APPENDIX

**Title :**Universal screening for hyperbilirubinemia in term healthy newborns at discharge: systematic review and meta-analysis

**Authors:** Faiza Khurshid, Suman Rao, Caroline Sauve, Shuchita Gupta

## Appendix S1 SEARCH STRATEGY

Last updated 31 Dec 2021

### Databases and search strategies

### Ovid Medline

| | | |
|---|---|---|
| 1 | Infant, Newborn/ | 639,527 |
| 2 | (newborn* or neonate* or infant*).tw,kf. | 638,125 |
| 3 | 1 or 2 | 985,486 |
| 4 | (visual adj5 (inspect* or assess* or observ* or screen* or evaluat*)).tw,kf. | 58,013 |
| 5 | Bilirubin/ | 24,911 |
| 6 | bilirubin*.tw,kf. | 41,776 |
| 7 | 5 or 6 | 50,459 |
| 8 | 4 and 7 | 98 |
| 9 | transcutaneous bilirubin*.tw,kf. | 465 |
| 10 | serum bilirubin*.tw,kf. | 6,845 |
| 11 | 8 or 9 or 10 | 7,096 |
| 12 | 3 and 11 | 2037 |

### EMBASE

| | | |
|---|---|---|
| 1 | Infant/ | 752626 |
| 2 | (newborn* or neonate* or infant*).tw,kw. | 807407 |
| 3 | 1 or 2 | 1245661 |
| 4 | (visual adj5 (inspect* or assess* or observ* or screen* or evaluat*)).tw,kw. | 81346 |
| 5 | Bilirubin/ | 96366 |
| 6 | bilirubin*.tw,kw. | 74930 |
| 7 | 5 or 6 | 118022 |
| 8 | 4 and 7 | 218 |
| 9 | transcutaneous bilirubin*.tw,kw. | 637 |

| | | |
|---|---|---|
| 10 | serum bilirubin*.tw,kw. | 11480 |
| 11 | 8 or 9 or 10 | 11907 |
| 12 | 3 and 11 | 2938 |

## COCHRANE CENTRAL

| | | |
|---|---|---|
| #1 | hyperbilirubinemia OR jaundice in Trials (Word variations searched) | 3630 |
| #2 | infant OR newborn OR neonate OR neonatal in Trials (Word variations searched) | 78871 |
| #3 | transcutaneous bilirubin OR TcB OR serum bilirubin OR total serum bilirubin OR TSB (Word variations searched) | 4737 |
| #4 | #1 AND #2 AND #3 in Trials (Word variations searched) 478 | 478 |

## CINAHL

| | | |
|---|---|---|
| S1 | (MH "Infant, Newborn") | 138,418 |
| S2 | TI infant* OR AB infant* | 115,973 |
| S3 | S1 OR S2 | 210,312 |
| S4 | TI (visual N5 (inspect* or assess* or observ* or screen* or evaluat*)) OR AB (visual N5 (inspect* or assess* or observ* or screen* or evaluat*)) | 14,755 |
| S5 | (MH "Bilirubin") | 3,022 |
| S6 | TI bilirubin* OR AB bilirubin* | 5,451 |
| S7 | S5 OR S6 | 6,386 |
| S8 | S4 AND S7 | 35 |
| S9 | TI transcutaneous bilirubin* OR AB transcutaneous bilirubin* | 209 |
| S10 | TI serum bilirubin* OR AB serum bilirubin* | 1,105 |
| S11 | S8 OR S9 OR S10 | 1,224 |
| S12 | S3 AND S11 | 569 |

## Web of Science

| | | |
|---|---|---|
| # 1 | 477645 | TOPIC: (infant*)<br>Indexes=SCI-EXPANDED, SSCI, A&HCI, ESCI Timespan=All years |

| | | |
|---|---|---|
| # 2 | 57,710 | TOPIC: ((visual NEAR/2 (inspect* or assess* or observ* or screen* or evaluat*) ))<br>Indexes=SCI-EXPANDED, SSCI, A&HCI, ESCI Timespan=All years |
| # 3 | 39,250 | TOPIC: (bilirubin*)<br>Indexes=SCI-EXPANDED, SSCI, A&HCI, ESCI Timespan=All years |
| # 4 | 95 | #3 AND #2<br>Indexes=SCI-EXPANDED, SSCI, A&HCI, ESCI Timespan=All years |
| # 5 | 523 | TOPIC: (transcutaneous bilirubin*)<br>Indexes=SCI-EXPANDED, SSCI, A&HCI, ESCI Timespan=All years |
| # 6 | 16,430 | TOPIC: (serum bilirubin*)<br>Indexes=SCI-EXPANDED, SSCI, A&HCI, ESCI Timespan=All years |
| # 7 | 16,650 | #6 OR #5 OR #4<br>Indexes=SCI-EXPANDED, SSCI, A&HCI, ESCI Timespan=All years |
| # 8 | 1,665 | #7 AND #1<br>Indexes=SCI-EXPANDED, SSCI, A&HCI, ESCI Timespan=All years |

## Grey literature

### Websites consulted

- Pediatric Academics Society Abstracts
  - Search terms used:
    - Jaundice
    - Bilirubin
- Belgian Health Care Knowledge Centre
  - Search terms used:
    - Jaundice
    - Bilirubin
- The Irish Health Repository
  - Search terms used:
    - Jaundice AND newborn*
    - Bilirubin AND newborn*
- National Institute for Health and Care Excellence
  - Search terms used:
    - Jaundice
- Agency for Healthcare Research and Quality
  - Search terms used:
    - Jaundice
    - Bilirubin
- Paediatric Economic Database Evaluation
  - Search terms used:
    - Jaundice AND bilirubin*
- Institute of Health Economics
  - Search terms used:
    - Bilirubin

- Academy of Medicine of Malaysia
    - Search terms used:
        - Jaundice
- Clinical Trials Registry – India
    - Search terms used:
        - Bilirubin
        - Newborn*
- ClinicalTrials.gov
    - Search terms used:
        - Hyperbilirubinemia, Neonatal
- PROSPERO
    - Search terms used:
        - Hyperbilirubinemia
        - Transcutaneous bilirubin*

## Fig S1 RISK OF BIAS IN INCLUDED STUDIES

A summary of the risk of bias assessment in the five included studies for comparison 1 is depicted in Figure 1a and 1b, and the risk of bias assessment for the three included studies for comparison 2 is depicted in Figure 2.

### Fig 1a. Risk of bias tables for comparison 1- Universal TcB vs. clinical screening- Randomized study (using RoB2)

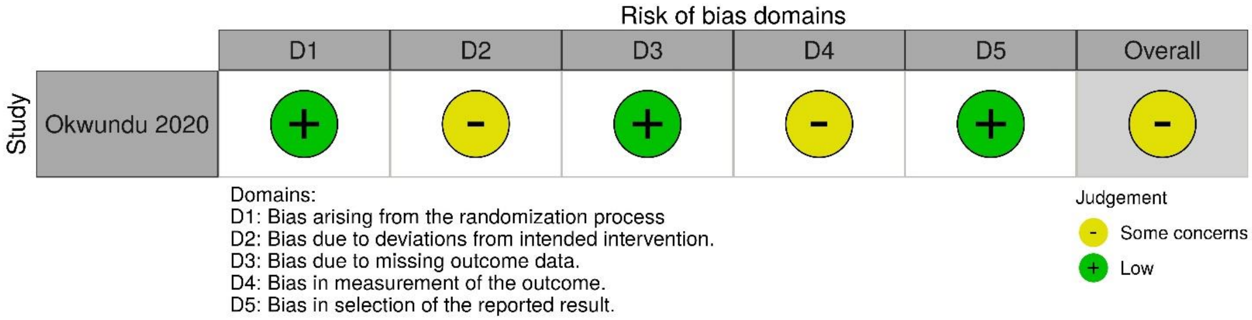


### Fig 1b. Risk of bias tables for comparison 1- Universal TcB vs. clinical screening- Non-randomized studies (using ROBINS-I)[1]

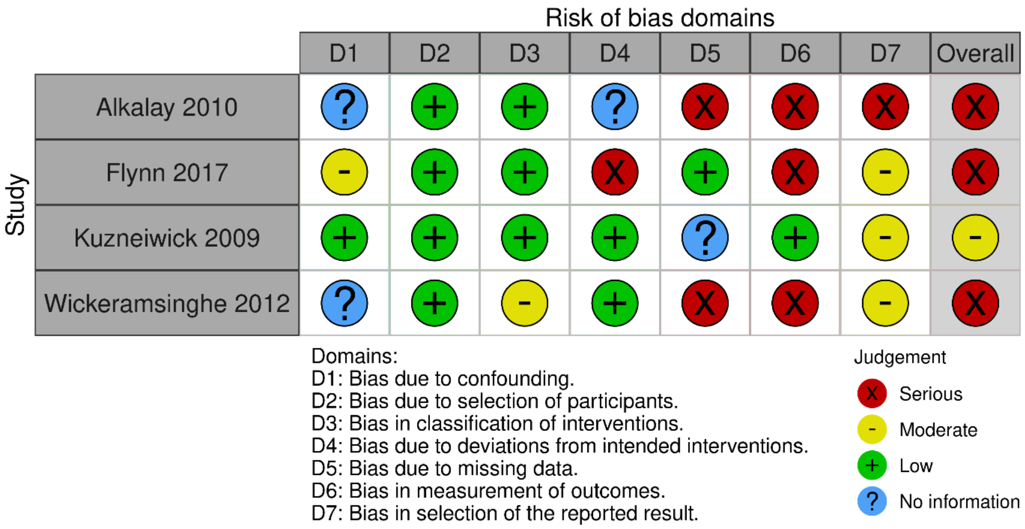

**Fig. S2. Risk of bias table for comparison 2: Universal TSB vs. clinical screening- Non-randomized studies (using ROBINS-I)[2]**

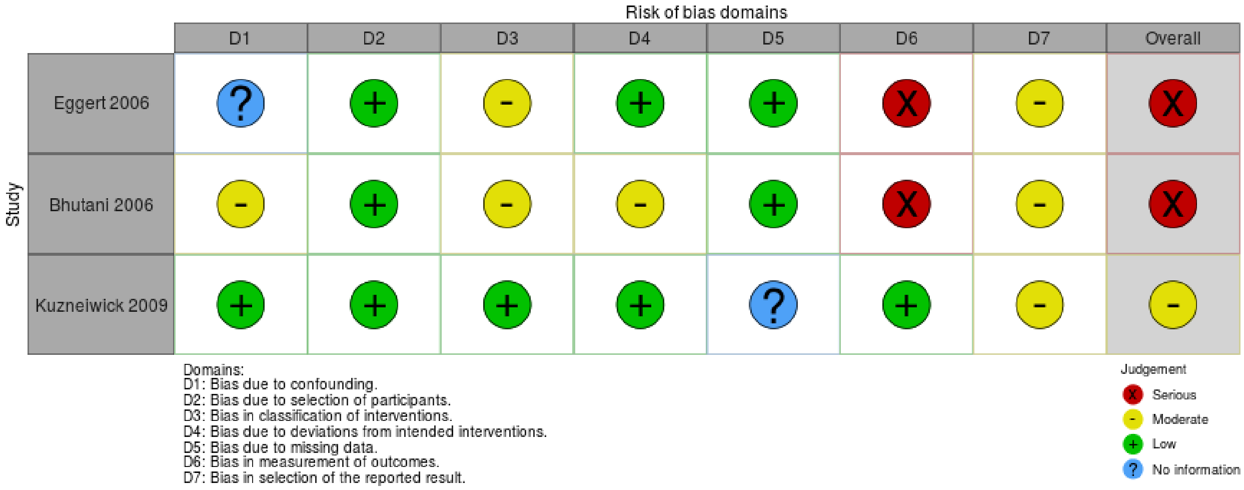

## Table S1 EXCLUDED STUDIES

**Table 1. Characteristics of excluded studies**

| Study | Reason for exclusion |
|---|---|
| Keren 2008[3] | No comparator group |
| O'Reilly 2015[4] | No comparator group (results compared to an earlier study) |
| Keren 2005[5] | Comparison of pre-discharge TSB is with predictive performance of a clinical risk factor scoring system |
| Kaplan 2008[6] | Did not report pre-specified outcomes |
| Mah 2010[7] | Evaluation of a combine program of universal screening where both TcB and TSB were implemented. The results are not provided separately by the type of screening (TcB and TSB). |
| Mishra 2009[8] | Included only visibly jaundiced neonates |
| Jonker 2016[9] | Included newborns with clinically observable jaundice |
| Allen NM 2010[10] | Not the right intervention. Clinical screening followed by TcB was compared with clinical screening followed by TSB. |
| Darling EK 2014[11] | Evaluates 2007 Canadian pediatrci Society (CPS) recommendations that recommended universal screening using either TSB or TcB, but does not provide the resuls separately by the type of screening (TcB and TSB) |
| Petersen 2005[12] | Visual inspection was compared with visual inspection followed by transcutaneous bilirubin (TcB; doneonly for newborns with clinically significant jaundice) |
| Morgan 2016[13] | Did not report pre-specified outcomes |
| Wainer 2012[14] | This study was not included as it was not restricted to pre-discharge TcB.<br><br>This study compared universal TcB with clinical screening using a before-and-after study design, enrolling 28908 newborns. The study included all healthy newborns ≥35 weeks gestation in a well-baby nursery and universal TcB in the post-implementation period (daily TcB in hospital and post-discharge in the community) with visual inspection by a public health nurse in the pre-implementation period. The study reported that universal TcB decreased severe hyperbilirubinemia (OR 0.45, 95% CI 0.31 to 0.65) and readmission for jaundice (OR 0.91, 95% CI 0.81 to 1.04) while the mean length of pre-discharge hospital stay remained the same (40.8±22.3 hours in universal TcB vs 40.3±21.5 hours in visual inspection group; MD 0.5 higher, 95% CI 0 to 1 higher). |
| Baker EK 2015[15] | Full text NA |

| | |
|---|---|
| Lacaze-Masmonteil 2012[16] | Conference abstract for the CT.gov trial protocol on community TcB (Abstracts: The 2011 Canadian Association of Paediatric Health Centres (CAPHC) Annual Conference); full-text NA |
| Bakhru 2018[17] | Full-text NA |
| Ur Rehman 2019[18] | Full text NA |

## Table S2 GRADE TABLES

.

## Table 2a. Comparison 1- Universal TcB vs. clinical screening

| Certainty assessment | | | | | | | № of patients | | Effect | | Certainty | Importance |
|---|---|---|---|---|---|---|---|---|---|---|---|---|
| **№ of studies** | **Study design** | **Risk of bias** | **Inconsistency** | **Indirectness** | **Imprecision** | **Other considerations** | **Universal TcB** | **Clinical screening** | **Relative (95% CI)** | **Absolute (95% CI)** | | |
| **Severe hyperbilirubinemia - RCT** | | | | | | | | | | | | |
| 1 | randomized trials | serious [a] | not serious | not serious | serious [b] | none | 3/929 (0.3%) | 11/929 (1.2%) | **RR 0.27** (0.08 to 0.97) | **9 fewer per 1000** (from 11 fewer to 0 fewer) | ⨁⨁◯◯ LOW | CRITICAL |
| **Severe hyperbilirubinemia - Non-RCTs** | | | | | | | | | | | | |
| 1 | observational studies | very serious [c] | not serious | not serious | not serious | none | - | - | **RR 0.25** (0.12 to 0.52) | **0 fewer per 1000** (from 1 fewer to 0 fewer) | ⨁⨁◯◯ LOW | CRITICAL |
| **Jaundice requiring exchange transfusion- RCT** | | | | | | | | | | | | |
| 1 | randomized trials | serious [a] | not serious | not serious | very serious [b,d] | none | 0/929 (0.0%) | 2/929 (0.2%) | **RR 0.20** (0.01 to 4.16) | **2 fewer per 1000** (from 2 fewer to 7 more) | ⨁◯◯◯ VERY LOW | CRITICAL |
| **Jaundice requiring exchange transfusion** – Non-RCTs | | | | | | | | | | | | |
| 1 | observational studies | serious [a] | not serious | not serious | not serious | none | - | - | OR 0.28 (0.19 to 0.42) | - | ⨁⨁◯◯ LOW | CRITICAL |
| **Bilirubin induced neurological dysfunction/Kernicterus-RCT** | | | | | | | | | | | | |
| 1 | randomized trials | serious [a] | not serious | not serious | very serious [b,d] | none | 0/929 (0.0%) | 1/929 (0.1%) | **RR 0.33** (0.01 to 8.17) | **1 fewer per 1000** (from 1 fewer to 8 more) | ⨁◯◯◯ VERY LOW | CRITICAL |
| **Readmission for jaundice - RCT** | | | | | | | | | | | | |
| 1 | randomized trials | serious [a] | not serious | not serious | serious[e] | none | 12/929 (1.3%) | 48/929 (5.2%) | **OR 0.24** (0.13 to 0.46) | **39 fewer per 1000** (from 45 fewer to 27 fewer) | ⨁⨁◯◯ LOW | CRITICAL |
| **Readmission for jaundice – Non-RCTs** | | | | | | | | | | | | |
| 4 | observational studies | very serious [c] | serious [f] | not serious | serious [d] | none | 55/8223 (0.7%) | 89/8266 (1.1%) | **OR 1.01** (0.38 to 2.70) | **0 fewer per 1000** (from 7 fewer to 18 more) | ⨁◯◯◯ VERY LOW | CRITICAL |

**CI:** Confidence interval; **RR:** Risk ratio; **OR:** Odds ratio, **RCT**: randomized controlled trial;

*Explanations*
a. Most of the pooled effect provided by studies at moderate risk of bias
b. Less than 30 events
c. Most of the pooled effect provided by studies at high risk of bias.
d. Wide confidence interval crossing the line of no effect.
e. Single study with sample size calculation based on 50% reduction with 80% power, downgraded considering the fragility of this result
f Statistical heterogeneity: I2 ≥60% or Chi2≤0.05

## Table 2b. Comparison 2- Universal TSB vs. clinical screening

| Certainty assessment | | | | | | | № of patients# | | Effect | | Certainty | Importance |
|---|---|---|---|---|---|---|---|---|---|---|---|---|
| **№ of studies** | **Study design** | **Risk of bias** | **Inconsistency** | **Indirectness** | **Imprecision** | **Other considerations** | **Universal TSB** | **Clinical screening** | **Relative (95% CI)** | **Absolute (95% CI)** | | |
| **Severe hyperbilirubinemia** | | | | | | | | | | | | |
| 2 | observational studies | very serious [a] | serious [b] | serious [c] | not serious | none | 370/52483 (0.7%) | 634/48798 (1.3%) | **OR 0.37** (0.15 to 0.88) | **8 fewer per 1,000** (from 11 fewer to 2 fewer) | ⊕◯◯◯ VERY LOW | CRITICAL |
| **Readmission for phototherapy** | | | | | | | | | | | | |
| 2 | observational studies | serious [d] | serious [b] | serious [c] | serious [e] | none | 226/52483 (0.4%) | 268/48798 (0.5%) | **OR 1.01** (0.62 to 1.67) | **0 fewer per 1,000** (from 2 fewer to 4 more) | ⊕◯◯◯ VERY LOW | CRITICAL |
| **Jaundice requiring exchange transfusion** | | | | | | | | | | | | |
| 2 | observational studies | serious [a] | serious [b] | serious [c] | serious [e] | none | 4/8549 (0.0%) | 13/22510 (0.1%) | **OR 0.53** (0.13 to 2.25) | **0 fewer per 1,000** (from 1 fewer to 1 more) | ⊕◯◯◯ VERY LOW | CRITICAL |

**CI:** Confidence interval; **OR:** Odds ratio
*# *No. of participants not reported by one study (Kuzneiwicz 2009), so the numbers shown are only from one study for each outcome*
*Explanations*
a. Most of pooled effect provided by studies at moderate or high risk of bias with >50% studies at high risk of bias.
b. Statistical heterogeneity: I2 ≥60% or Chi2≤0.05.
c. The studies enrolled preterm newborns (≥35 weeks) and they did not specify the proportion.
d. Most of pooled effects provided by studies at moderate or high risk of bias with <50% studies at high risk of bias.
e. Wide CI crossing the line of no effect.

## References

[1] Sterne JAC, Hernán MA, Reeves BC, Savović J, Berkman ND, Viswanathan M, Henry D, Altman DG, Ansari MT, Boutron I, Carpenter JR, Chan AW, Churchill R, Deeks JJ, Hróbjartsson A, Kirkham J, Jüni P, Loke YK, Pigott TD, Ramsay CR, Regidor D, Rothstein HR, Sandhu L, Santaguida PL, Schünemann HJ, Shea B, Shrier I, Tugwell P, Turner L, Valentine JC, Waddington H, Waters E, Wells GA, Whiting PF, Higgins JPT. ROBINS-I: a tool for assessing risk of bias in non-randomized studies of interventions. BMJ 2016; 355; i4919; doi: 10.1136/bmj.i4919

[2] Sterne JAC, Hernán MA, Reeves BC, Savović J, Berkman ND, Viswanathan M, Henry D, Altman DG, Ansari MT, Boutron I, Carpenter JR, Chan AW, Churchill R, Deeks JJ, Hróbjartsson A, Kirkham J, Jüni P, Loke YK, Pigott TD, Ramsay CR, Regidor D, Rothstein HR, Sandhu L, Santaguida PL, Schünemann HJ, Shea B, Shrier I, Tugwell P, Turner L, Valentine JC, Waddington H, Waters E, Wells GA, Whiting PF, Higgins JPT. ROBINS-I: a tool for assessing risk of bias in non-randomized studies of interventions. BMJ 2016; 355; i4919; doi: 10.1136/bmj.i4919

[3] Keren R, Luan X, Friedman S, Saddlemire S, Cnaan A, Bhutani VK. A comparison of alternative risk-assessment strategies for predicting significant neonatal hyperbilirubinemia in term and near-term infants. Pediatrics. 2008 Jan;121(1):e170-9. doi: 10.1542/peds.2006-3499. PMID: 18166536.

[4] O'Reilly P, Walsh O, Allen NM, Corcoran JD. The Bhutani Nomogram Reduces Incidence of Severe Hyperbilirubinaemia in Term and Near Term Infants. Ir Med J. 2015 Jun;108(6):181-2. PMID: 26182804.

[5] Keren R, Bhutani VK, Luan X, Nihtianova S, Cnaan A, Schwartz JS. Identifying newborns at risk of significant hyperbilirubinaemia: a comparison of two recommended approaches. Arch Dis Child. 2005 Apr;90(4):415-21. doi: 10.1136/adc.2004.060079. PMID: 15781937; PMCID: PMC1720335.

[6] Kaplan M, Shchors I, Algur N, Bromiker R, Schimmel MS, Hammerman C. Visual screening versus transcutaneous bilirubinometry for predischarge jaundice assessment. Acta Paediatr. 2008 Jun;97(6):759-63. doi: 10.1111/j.1651-2227.2008.00807.x. PMID: 18460107.

[7] Mah MP, Clark SL, Akhigbe E, Englebright J, Frye DK, Meyers JA, Perlin JB, Rodriguez M, Shepard A. Reduction of severe hyperbilirubinemia after institution of predischarge bilirubin screening. Pediatrics. 2010 May;125(5):e1143-8. doi: 10.1542/peds.2009-1412. Epub 2010 Apr 5. PMID: 20368324.

[8] Mishra S, Chawla D, Agarwal R, Deorari AK, Paul VK, Bhutani VK. Transcutaneous bilirubinometry reduces the need for blood sampling in neonates with visible jaundice. Acta Paediatr. 2009 Dec;98(12):1916-9. doi: 10.1111/j.1651-2227.2009.01505.x. Epub 2009 Oct 7. PMID: 19811459.

[9] van den Esker-Jonker B, den Boer L, Pepping RM, Bekhof J. Transcutaneous Bilirubinometry in Jaundiced Neonates: A Randomized Controlled Trial. Pediatrics. 2016 Dec;138(6):e20162414. doi: 10.1542/peds.2016-2414. Epub 2016 Nov 4. PMID: 27940715.

[10] Allen NM, O'Donnell SM, White MJ, Corcoran JD. Initial assessment of jaundice in otherwise healthy infants--a comparison of methods in two postnatal units. Ir Med J. 2010 Nov-Dec;103(10):310-3. PMID: 21560504.

[11] Darling EK, Ramsay T, Sprague AE, Walker MC, Guttmann A. Universal bilirubin screening and health care utilization. Pediatrics. 2014 Oct;134(4):e1017-24. doi: 10.1542/peds.2014-1146. Epub 2014 Sep 22. PMID: 25246625.

[12] Petersen JR, Okorodudu AO, Mohammad AA, Fernando A, Shattuck KE. Association of transcutaneous bilirubin testing in hospital with decreased readmission rate for hyperbilirubinemia. Clin Chem. 2005 Mar;51(3):540-4. doi: 10.1373/clinchem.2004.037804. PMID: 15738516.

[13] Morgan MC, Kumar GS, Kaiser SV, Seetharam S, Ruel TD. Implementation of a neonatal transcutaneous bilirubin screening programme in rural India. Paediatr Int Child Health. 2016 May;36(2):122-6. doi: 10.1179/2046905515Y.0000000013. PMID: 25844503.

[14] Wainer S, Parmar SM, Allegro D, Rabi Y, Lyon ME. Impact of a transcutaneous bilirubinometry program on resource utilization and severe hyperbilirubinemia. Pediatrics. 2012 Jan;129(1):77-86. doi: 10.1542/peds.2011-0599. Epub 2011 Dec 19. PMID: 22184646.

[15] Baker EK, Sweetman DU, Jacobs SE, Owen LS. Does pre-discharge newborn transcutaneous bilirubinometry (tcb) screening reduce blood sampling and presentation to emergency services with hyperbilirubinaemia?  Journal of Paediatrics and Child Health 51 (Suppl. 1) (2015), 1–138 © 2015 The Authors. Paediatrics and Child Health Division (Royal Australasian College of Physicians)

[16] Thierry Lacaze-Masmonteil, Janie Tyrrell, Cathy Kimak, Heather Chinnery, Rick Watts, Nancy Bott, George Blakney, Selikke Janes-Kelley, Phil Etches, Rhonda J. Rosychuk. PO2.103. The Use of Transcutaneous Bilirubinometry for Monitoring Jaundiced Neonates in the Community Reduces the Need for Blood Sampling with no Increased Risk for Significant Hyperbilirubinemia. The 2011 Canadian Association of Paediatric Health Centres (CAPHC) Annual Conference
Abstracts. Ottawa, Ontario. October 16 - 19, 2011

[17] V Bakhru, R Dara, J Bakhru and S Choudhary. P-667. Universal bilirubin screening: a new hope to limit exchange transfusion in severe hyperbilirubinemia. Abstract in International Journal of Transfusion Medicine

[18] Liqa ur Rehman, Mushtaq Hussein, Naeem Shori, Riaz Ahmed, Muhammad Zia, Muhammad Azam. Effectiveness and reliability of pre-discharge jaundice assessment tools: A quality improvement project (10.1136/archdischild-2019-epa.497)
